# Electrospun Nanofiber-Based Bioinspired Artificial Skins for Healthcare Monitoring and Human-Machine Interaction

**DOI:** 10.3390/biomimetics8020223

**Published:** 2023-05-26

**Authors:** Xingwei Chen, Han Li, Ziteng Xu, Lijun Lu, Zhifeng Pan, Yanchao Mao

**Affiliations:** Key Laboratory of Materials Physics of Ministry of Education, School of Physics and Microelectronics, Zhengzhou University, Zhengzhou 450001, China

**Keywords:** artificial skin, electrospun nanofiber, healthcare monitoring, human-machine interaction

## Abstract

Artificial skin, also known as bioinspired electronic skin (e-skin), refers to intelligent wearable electronics that imitate the tactile sensory function of human skin and identify the detected changes in external information through different electrical signals. Flexible e-skin can achieve a wide range of functions such as accurate detection and identification of pressure, strain, and temperature, which has greatly extended their application potential in the field of healthcare monitoring and human-machine interaction (HMI). During recent years, the exploration and development of the design, construction, and performance of artificial skin has received extensive attention from researchers. With the advantages of high permeability, great ratio surface of area, and easy functional modification, electrospun nanofibers are suitable for the construction of electronic skin and further demonstrate broad application prospects in the fields of medical monitoring and HMI. Therefore, the critical review is provided to comprehensively summarize the recent advances in substrate materials, optimized fabrication techniques, response mechanisms, and related applications of the flexible electrospun nanofiber-based bio-inspired artificial skin. Finally, some current challenges and future prospects are outlined and discussed, and we hope that this review will help researchers to better understand the whole field and take it to the next level.

## 1. Introduction

The advancement of wearable electronics has been attracting more and more attention recently due to their ability to simulate the haptic perception of human skin to identify changes in detected external information through different electrical signals [1,2,3,4,5]. Unlike traditional rigid electronic devices that cannot maintain polymorphic contact with the human body, wearable electronic products can serve for health management or providing other smart functions, which greatly enrich people’s daily needs. Among them, bioinspired artificial skin is considered to be an important component of wearable electronic devices that can be affixed to the surface of human muscles or joints to collect physiological signals, with promising applications in the areas of real-time healthcare monitoring and human-machine interaction (HMI) [6,7,8,9,10,11,12,13]. Therefore, the design of artificial skin needs to be considered on skin-like flexible materials, mainly focusing on the stable monitoring of artificial skin in use, wearing comfort, and physical and chemical properties suitable for human skin. Electrospinning-based flexible devices provide a practical path for human skin construction based on such flexible material substrates [14,15,16,17,18].

Typically, electrospinning is a particular method of fiber manufacturing that uses a solution or melt of polymer for jet spinning under a high voltage electric field, which produces nanometer diameter polymer fibers with flexibility and continuity. The idea of electrospinning was conceived in 1600 by William Gilbert, who observed in his research that water droplets would form cones in an electric field [19]. In 1887, Charles V. Boys used a viscous liquid to pull out fibers while on the edge of an insulated dish connected to a power source, and the method of extracting fibers from a viscoelastic liquid under strong electric field conditions was first reported. In 1902, the electrospinning technology was patented by John Cooley and William Morton, respectively, and the prototype of the electrospinning device was determined [20]. From 1964–1969, a number of papers were published by Jeffrey Taylor, mathematically describing and simulating the process of changing a viscous polymer solution from a sphere to a cone at an electrospinning nozzle under the effects of a high-voltage electric field, achieving a breakthrough of electrospinning technology [21,22,23,24]. However, electrospinning technology development has stalled because of the absence of microscopic-scale characterization tools. It was not until the beginning of this century with the popularization of electron microscopy that the technology began to receive more and more attention from researchers, and the performance and applications were developed as never before. Through the process of developing new strategies to control structures and performances of electrospun nanofibers, electrospinning technology had already been used extensively in the area of bio-inspired artificial skin.

Artificial skin, as bionic human skin, needs to meet the characteristics of the high elasticity and breathability of human skin [25]. In the electrospinning process, polymer solution jets are stretched in a strong electric field to form nanofibers ranging from a few nanometers to 500 nanometers in diameter, which are then deposited on a collection plate to form a nanofiber film [26,27,28]. Compared with the thin film type flexible substrate material, the mesh structure of the nanofiber membrane makes it flexible and breathable, with a great ratio of surface area and thermal stability, which can better meet the material requirements of artificial skin [29,30,31,32,33]. The performance of electrospun nanofibers can be further enhanced by adding different nano-fillers to develop artificial skin with different structures and different functions [34,35,36,37,38,39].

**Figure 1:**
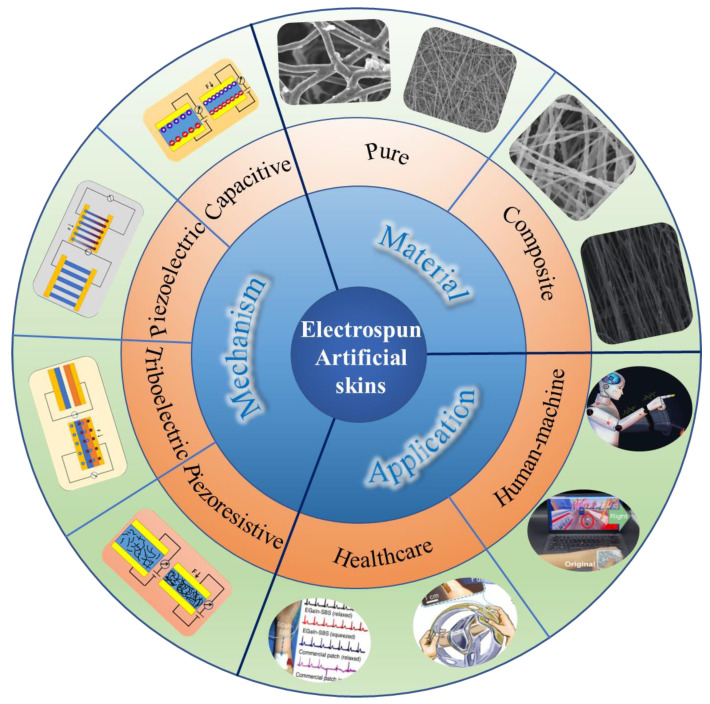
Summary of electrospun-based bionic artificial skin, which includes the material selection, working mechanism, and related applications.

In this review, we aim to present a complete overview of electrospun nanofiber-based bioinspired artificial skin for healthcare monitoring and HMI. We begin with an introduction to the apparatus and principles of electrospinning to give the reader a basic understanding of electrospinning technology. Then, as shown in Figure 1, we summarize the materials that can be used for electrospinning, reviewing pure organic polymer solutions and composites incorporated with nanofillers for electrospinning, respectively. Afterward, we discuss four common sensing mechanisms for artificial skin devices, including sensing principles, application areas, and advantages and disadvantages. Moreover, we also focus on reviewing and highlighting the recent research advances in human healthcare monitoring and HMI based on electrospun nanofibers for bionic artificial skin. Finally, after a simple summary, we discuss new directions for the development of bionic artificial skin and the challenges that need to be faced in the future. This review is expected to help researchers acquire more comprehensive understanding of the area to then advance it to the next level.

## 2. Electrospinning Method

There are many methods producing fibers from synthetic polymers such as dry spinning, gel spinning, melt spinning, wet spinning, etc. [40]. However, limited by equipment and technology, the diameter of the extracted fibers could not reach the submicron level, which seriously restricts their further application. It was not until Charles V. Boys proposed the method of extracting fibers from viscoelastic liquids under high voltage in 1887 that electrospinning technology received widespread attention from researchers and was further developed. So far, the current electrospinning technology can produce very fine diameter fibers, from a few nanometers to a few microns, and even less than 1 nanometer [41]. Fibers less than 500 nm in diameter prepared by electrospinning are often referred to as nanofibers, and this fabrication technology can be used from different scientific and engineering aspects. The lightweight and porous characteristics of flexible electrospinning materials make it very suitable for the research of multi-functional materials, such as flexible devices for bionic artificial skin, etc.

**Figure 2:**
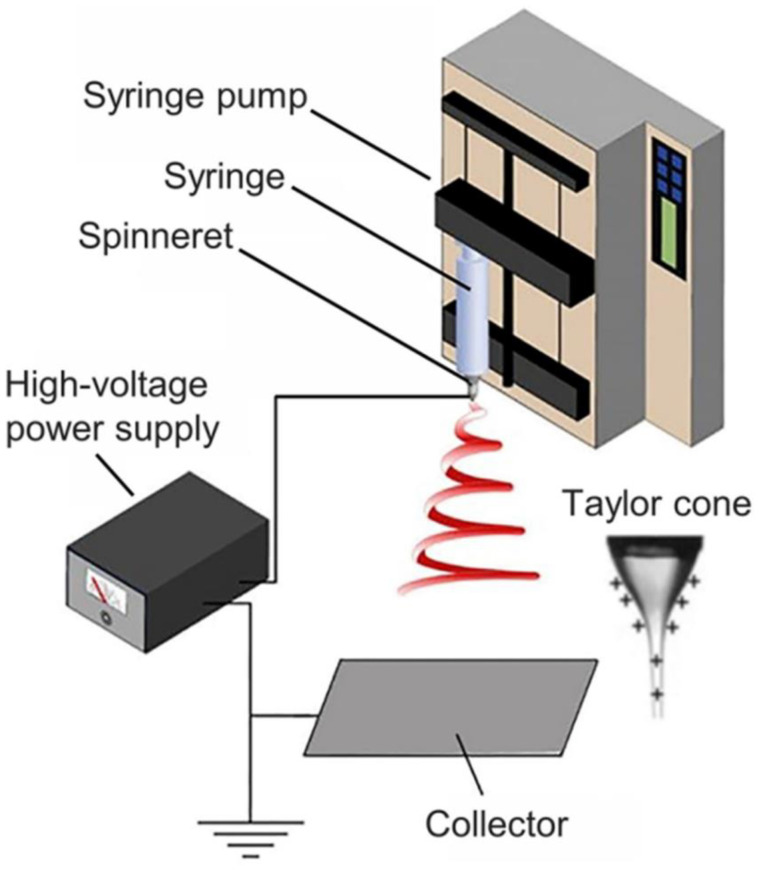
Illustration of electrospinning apparatus [23]. Copyright 2017 American Chemical Society.

As illustrated in Figure 2, the major components of the electrospinning device include a conductive collector, a syringe pump, a syringe, a spinneret, a high voltage power source, etc. [23,42]. In the preparation process, a polymer precursor solution with the appropriate concentration, viscosity, and conductivity is transferred into the syringe, and the syringe tip is connected to the high voltage power supply, so that droplets flowing from the needle form a Taylor cone by electrostatic action. When the electric field strength is further enhanced by increasing the voltage, the Taylor cone will generate charged jets and be stretched to a certain extent due to bending instability. In this process, the jet will become longer and thinner with the specific surface area greatly increasing, which also accelerates the volatilization of the solvent [43]. Therefore, it will solidify rapidly and finally deposit nanofibers on the collecting plate. Following deposition, the bulk of the charge on the fibers is rapidly dissipated through a grounded conductive collector. However, owing to the low conductivity of the general polymer material, there will still be part of the charge remaining on the fiber surface, which will lead to mutual repulsion with other jets of the identical charge. Therefore, electrospinning nanofiber films can only reach a very thin thickness, typically less than 1 mm.

Electrospinning involves electrohydrodynamic processes in which droplets are charged to produce a jet [23]. When the electric field force on the solution reaches a critical value that overcomes the apparent tension, the Taylor cone ejects charged jets in the direction along the cone head, and then stretches under an electric field to form nanofibers. In this process, the critical voltage *V* is calculated according to the following formula:

(1) $$V=\frac{4H^{2}}{L^{2}}[\ln(\frac{2L}{R})-1.5](1.3\pi R\gamma )(0.09)$$

where *L* is the length of the jet, *H* is the distance among the electrodes, *R* is the radius of the syringe nozzle, and *γ* is the apparent tension of the polymer solution. The Taylor cone ejects a charged jet initially moving in a direct line, and the length of the direct line can be calculated by:

(2) $$L=\frac{4kQ^{2}}{\pi \rho^{2}I^{2}}\{{\left[{\left(\frac{2\sigma Q}{\pi k\rho E}\right)}^{\frac{1}{3}}\right]}^{2}-r_{0}{}^{2}\}$$

where *k* is the solution electrical conductivity, *Q* is the propulsion speed of the propulsion pump, *I* is the electric current flowing across the jet, *ρ* is the solution density, *r*_0_ is the original radius of the jet, *σ* is the surface charge of the jet, and *E* is the strength of the electric field. Different experimental parameters or spinning solutions require different critical voltages to generate the jet, and the linear motion length of the jet is an important guide to set the distance between the nozzle and the collector.

During the electrospinning process, the processing parameters directly impact the structure, shape, and properties of the fabricated nanofibers. Processing parameters mainly include factors such as the electrospinning solution, electrospinning process, temperature, and humidity during electrospinning. On one hand, the properties of the electrospinning solution are governed by the molecular weight and molecular weight density distribution of polymer. On the other hand, this is also closely related to the viscosity, conductivity, and surface tension of the polymer solution along with the volatility and conductivity of the solvent. In addition, the effect of adding surfactants and inorganic salts on the polymer blend solution should be considered. The key to the formation of a polymer solution suitable for electrospinning lies in the solubility parameters of a suitable solvent [44]. However, besides the high solubility parameter, the volatility of the solvent also affects the curing speed of the spray and has an impact on the formation of the fiber surface structure. When the solvent volatility is fast, the fibers form a smooth surface as the high volatility may lead to immediate solidification after the jet. On the contrary, when the volatility is low, it is hard to form micropores on the surface of the fibers, which leads to a rougher fiber surface. Too low of a volatility can lead to spinning failure because the fibers remain in a liquid state when deposited on the collection plate.

Additionally, if the discharge constant of the solvent is too large, it will lead to a greater electrostatic rejection between the charges on the surface of the jet, and larger voltage, will be required to stabilize the jet, which is also not conducive to electrospinning. Commonly used solvents for electrospinning include acetone, dimethylformamide, alcohol, methylene chloride, tetrahydrofuran, etc. Electrospinning process parameters mainly include electric field intensity, spinning fluid flow, etc. The electric field strength depends on the voltage, and only when the voltage reaches a threshold value, the Taylor cone can emit a jet which eventually forms a fiber. The magnitude of the imposed voltage can directly determine the magnitude of the charge that the jet carries and the electrostatic rejective force between surface charges. Usually, a higher voltage is more conducive to the formation of finer fibers. The velocity of the spinning solution flow has some subtle effects on the diameter of the fiber. Usually, increasing the speed causes the formation of larger diameter nanofibers, but in different solution systems, the pattern is not exactly the same. The environment during electrospinning, which includes the environmental temperature and relative humidity, is also significant to the formation of fibers. The increase in temperature reduces the adhesive force and apparent tension of the polymer solution, which facilitates finer fiber formation. However, too high of a temperature hastens the evaporation of the solvent, which limits the length of the spray extension. Therefore, the temperature should be controlled in an appropriate range during electrospinning. Similarly, when the relative humidity is low, it facilitates the formation of fine fibers with dry surfaces, but too low a relative humidity will result in excessive evaporation in the jet which affects the jet’s extension length [45]. Both the temperature and relative humidity have two-sided effects on electrospinning and need to be adjusted according to the situation.

Electrospun nanofibers are broadly used to construct flexible bioinspired artificial skin due to its high ratio surface area, unique porous network structure, and stability [46]. With the benefit of a high relative surface area, especially the insensitivity and excellent elasticity of nanofiber membranes to mechanical deformation, the resulting sensing device can have improved detection capabilities, making it a good e-skin candidate for the field of healthcare monitoring and HMI.

## 3. Materials

Electrospinning technology has now been utilized in various fields. Different kinds of materials can be used to prepare nanofibers, such as most natural or synthesized organic polymers, which could be used directly for electrospinning once they are soluble in a suitable solvent. Generally, it is usually necessary that the small molecules self-assemble and produce sufficient chain entanglement or chemical binding to the solution-gel. In addition, by doping polymer solutions with additives such as nanoparticles, nanotubes, nanosheets, and other nanofibers, the resulting hybrid solutions can also be electrospun [47,48,49,50,51,52,53,54].

### 3.1. Pure Polymer Nanofibers

**Figure 3:**
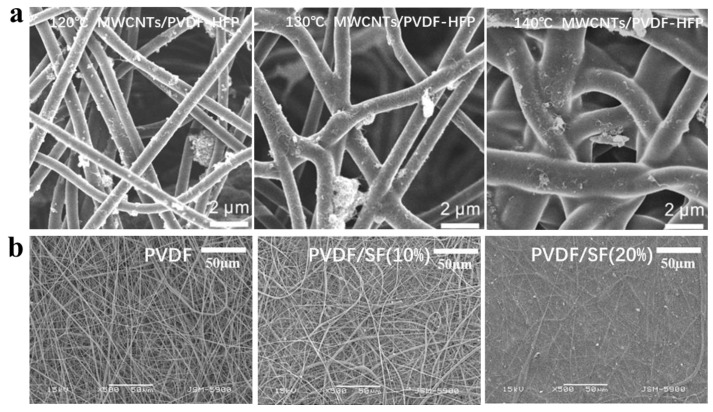
(**a**) SEM images of PVDF-HFP nanofibers with MWCNTs added after annealing treatment under 120, 130, and 140 °C for ten minutes, respectively [86]. Copyright 2019 American Chemical Society. (**b**) SEM images of PVDF/SF fibers added with 0%, 10%, and 20% filament protein nanofibers [87]. Copyright 2021 John Wiley & sons.

In general, electrospun polymer solutions require dissolved polymers with a sufficiently high molecular weight and a suitable solvent, because if the molecular weight is not high enough, chain entanglement is limited and electrospinning would generate beads instead of fibers. Among the more than one hundred polymers that for solution electrospinning can be used directly, common polymers include polycaprolactone (PCL) [55], polylactic acid (PLA) [56,57,58,59], polyaniline (PANI) [60,61,62,63], polypyrrole (PPy) [64,65], DNA [66], poly (lactic-co-glycolic acid) (PLGA) [67], Polyvinyl alcohol (PVA) [68,69,70,71,72,73,74,75,76,77], and gelatin are widely used in the manufacturing of biomedical scaffolds [78]. What is more, synthetic polymers are also often used in electrospinning, such as polystyrene (PS), and polyvinyl chloride (PVC) are used in areas related to environmental protection and monitoring. Interestingly, polyvinylidene fluoride (PVDF) and its copolymers can be also used for electrospun nanofibers and have good performance in the field of artificial skin and flexible sensing [79,80,81,82,83,84,85]. For example, Huang et al. proposed a strategy of nanofiber mats with conductivity for wearable devices by first coating MWCNTs on electrospun polyvinylidene fluoride-hexafluoropropylene (PVDF-HFP) fibers and then embedding them on the PVDF-HFP nanofiber surface using a further thermal welding process to obtain both breathable and conductive MWCNTs/PVDF-HFP nanofiber mats [86], which have excellent application prospects in the area of wearable electronic devices. Figure 3a shows the SEM images of PVDF-HFP nanofibers after ten minutes of thermal annealing treatment with MWCNTs at 120, 130, and 140 °C environments, respectively. It can be clearly observed that PVDF-HFP fibers did not reach the melting point and no fusion with MWCNTs occurred at 120 °C. The nanofibers started to fuse at 130 °C, and the fusion effect between fibers was more obvious at 140 °C. However, excessive fusion also results in a significant decrease in the permeability of the fiber mat. In addition to using thermal treatment methods to improve the performance of electrospun nanofiber mats, undoped nanofiller hybrid types of polymeric electrospun composite nanofiber mats can be fabricated by arranging coaxial nozzles or using nozzles facing the collector. Cheol Sang Kim et al. made electrospun PVDF and filament protein (SF) nanofibers into composite fiber mats by placing two nozzles facing each other’s mats [87]. Figure 3b shows the SEM images of the composite fiber mats at 0%, 10%, and 20% SF nanofiber ratios. Through piezoelectric tests and tensile experiments, this electrospun nanofiber composite mat exhibits better mechanical properties and biocompatibility than a single material, showing a potential use of electrospinning technology in the area of flexible sensing relevant to human healthcare monitoring.

### 3.2. Polymer Composite Nanofibers Incorporated with Nanofillers

**Figure 4:**
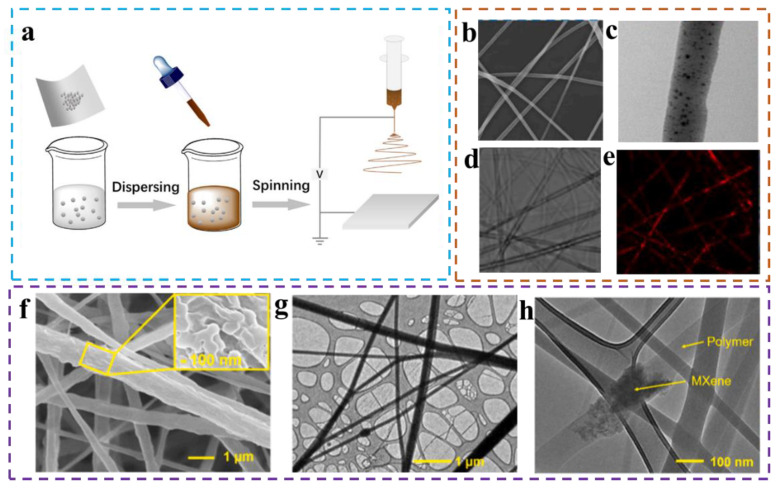
(**a**) Schematic illustration of electrospinning of solution doped with additives [97]. Copyright 2018 Elsevier. FE-SEM image (**b**), TEM image (**c**), (**d**,**e**) confocal fluorescent spectrum of PPNG pad with 1 *v*/*v*% IPQDs added [95]. Copyright 2021 Elsevier. (**f**) FE-SEM image of PMC nanofibers (inset shows the roughness on the fibers at the nanoscale). (**g**,**h**) TEM images of PMC nanofibers at different scales, showing the insertion of MXene nanosheets in PVDF substrate [96]. Copyright 2020 Elsevier.

Usually, to enhance the properties of electrospun nanofibers, different kinds and concentrations of conductive and non-conductive fillers are doped into the organic polymer solution to be electrospun (Figure 4a) [88,89,90,91,92,93,94]. For example, Kuo et al. presented a flexible electrospun optoelectronic device by mixing the inorganic chalcogenide quantum dots and cellulose nanocrystal composites (IPQDs/CNC) into a PVDF solution for the next electrospinning process [95]. Figure 4b–e show FE-SEM images, TEM images, and the confocal fluorescent spectrum of these composite nanofibers made from a hybrid electrospinning solution with an IPQDs doping content of 1 *v*/*v*%. The addition of IPQDs leads to improved electrical conductivity and the adhesion of the mixed electrospinning solution, and the resulting nanofibers were slender and smoother. The improved piezoelectric properties of the composite nanofiber devices can be ascribed to the phase change of the crystal structure. Electrospun composite nanofibers are not only used to make piezoelectric sensors, but also triboelectric sensors. Park et al. firstly proposed to blend MXene (Ti_3_C_2_T_x_) nanosheets into a PVDF matrix [96], and the resulting hybrid material (PVDF/MXene composite, PMC) was electrospun and used for the negative layer of triboelectric nanogenerators (TENG). Figure 4f shows FE-SEM images of PMC nanofibers; Figure 4g and h show TEM images of PMC nanofibers at different scales, indicating that MXene nanosheets have been successfully embedded into the PVDF matrix. Compared with the pure PVDF nanofiber and nylon nanofiber anodes, PMC nanofibers exhibited excellent performance in terms of permittivity and surface charge density, with an increase of 270% and 80%, respectively.

**Figure 5:**
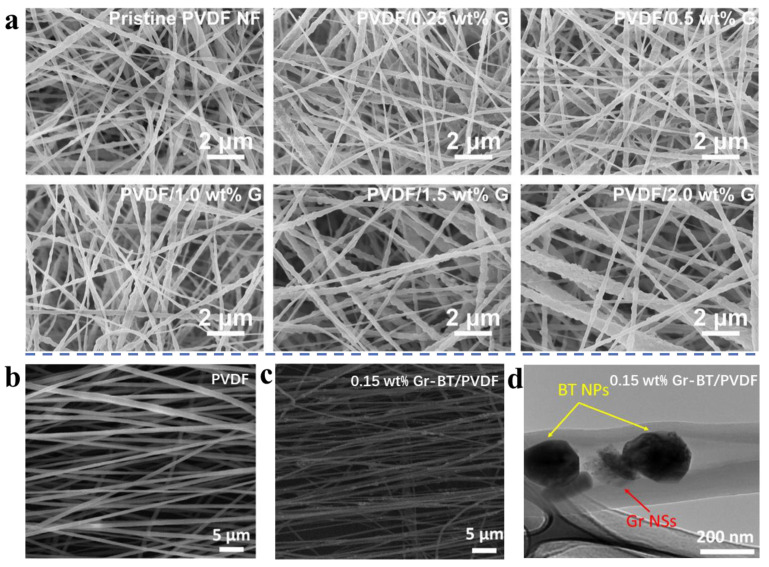
(**a**) SEM images for different GNS density (0–2.0 wt%) of electrospun PVDF/G composite nanofibers [98]. Copyright 2020 Elsevier. (**b**) SEM image of pure PVDF fibers. (**c**) SEM image of Graphene-BT/PVDF nanocomposite fibers with graphene concentration of 0.15 wt%. (**d**) TEM image of Graphene-BT/PVDF nanocomposite fibers with graphene concentration of 0.15 wt% [99]. Copyright 2018 Elsevier.

Among the reported hybrid solutions for electrospun nanofibers, graphene nanosheets are one of the most popular nanofiller choices for enhancing the conductivity of composite nanofibers. In 2020, Luo et al. proposed a new strategy of electrospinning composite nanofibers based on PVDF and graphene nanosheets (GNSs) for TENGs by adding GNSs to the electrospun precursor solution with pre-optimized PVDF for magnetic stirring [98]. Compared to pure PVDF nanofibers, the composite nanofibers made by electrospinning have a smoother fiber surface with denser pores, and the fiber morphology did not change significantly with the increase of GNSs concentration (Figure 5a). Interestingly, a hybrid PVDF-based electrospinning strategy developed by Jiang et al. has been previously reported to construct piezoelectric nanogenerators by doping with GNSs. [99]. However, the difference is that they also added barium titanate nanoparticles (BaTiO_3_). Figure 5b–d show the SEM images of PVDF fibers, 0.15 wt% Gr-BT/PVDF composite nanofibers, and TEM images, respectively. The pure PVDF fiber has a smooth surface, while the surface morphology of the composite nanofibers is rough, as graphene nanosheets and BaTiO_3_ nanoparticles are incorporated into the fibers. The piezoelectric performance of the composite nanofiber mat was significantly improved due to the synergistic effect of BaTiO_3_ and GNSs.

## 4. Working Mechanism

**Figure 6:**
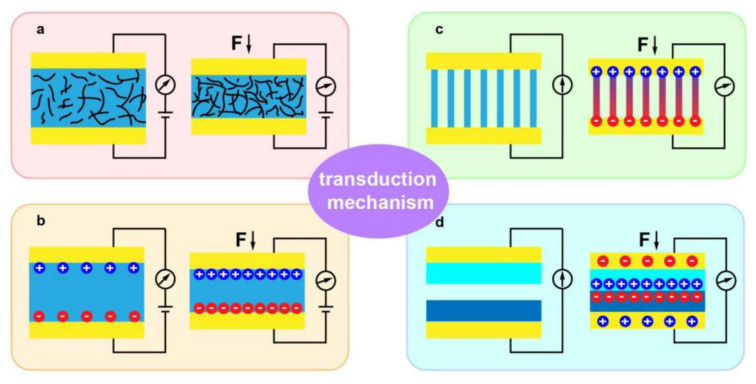
The illustration of four typical sensing mechanisms: (**a**) piezoresistive, (**b**) capacitive, (**c**) piezoelectric, (**d**) triboelectric sensing [104]. Copyright 2021 MDPI.

To date, the vast majority of reported artificial skin sensors work by a mechanism that transforms external mechanical stimuli into electrical signals, which can be understood as the ability to perceive external forces such as pressure, shear, strain, and distortion deformation, etc. The main operating principles are piezoresistive [100], capacitive [101], piezoelectric [102], and triboelectric effects [103]. Figure 6 shows four typical sensing mechanisms in the area of flexible electronics [104]. Depending on the mechanism of sensing, flexible sensors can be classified as piezoresistive, capacitive, piezoelectric, and triboelectric sensors [105,106,107]. Each of these four types has its own unique characteristics.

### 4.1. Piezoresistive Effect

The piezoresistive sensor is designed based on the piezoresistive effect and it was first used commercially. When pressure or strain is exerted externally, the resistance of the sensor changes. Traditional piezoresistive sensors have poor flexibility and ductility, and can only measure strain in a specific direction. In recent years, researchers have been devoted to developing innovative materials that are stretchable, highly sensitive, and flexible for making piezoresistive sensors for human motion detection, artificial skin, and other applications. For example, many piezoresistive composite sensors are formed by embedding conductive fillers such as carbon black, gold nanoparticles (AuNPs), silver nanoparticles (AgNPs), etc., into flexible electrospun nanofibers. What is more, conductive fillers with the same role include metal nanowires, carbon nanotubes (CNTs), graphene, and Ag nanosheets, which are also selected to be doped into the flexible substrates such as polydimethylsiloxane (PDMS), polyurethane (PU), hydrogels, etc. [108,109]. Therefore, conductive polymer composites are considered to be the most promising flexible piezoresistive sensors due to their wider choice of materials and structural design.

### 4.2. Capacitive Effect

Capacitive sensors are usually made of a flexible medium layer sandwiched between two electrodes. The change of capacitance is affected not only by the external pressure magnitude, but also by the parallel plate electrodes’ relative position to each other, which has the advantages of high sensitivity, lower energy consumption, as well as good static detection capability. The main disadvantage is that negative effects caused by parasitic capacitance accumulated during use [110]. The preparation method of flexible capacitive sensors mainly uses metal films as electrodes, and the elastomer was sandwiched between the electrode plates as dielectric layers. Generally, low-modulus elastic dielectrics include PDMS, Ecoflex, acrylic elastomers, etc., and are commonly employed. However, the sensitivity and reaction time of capacitive sensors are usually not particularly good due to the inherent viscoelasticity of elastic dielectrics. To improve the capacitive sensor sensitivity, elastomeric dielectrics can choose dielectric materials with micro/nanostructures, and the electrospun nanofibers are favorable candidates. Usually materials with micro/nanostructures can also produce a large deformation at low pressure, thus achieving the purpose of improving sensor sensitivity.

### 4.3. Piezoelectric Effect

Piezoelectric sensors are based on dipole-polarized piezoelectric materials that produce a voltage due to a change in potential under an applied strain, allowing a transformation of mechanical stimuli into electrical signals, which has the advantage of high sensitivity and stability. However, the disadvantage is that it is not suitable for measuring static pressure signals and there are some limitations in low frequency detection, so it is widely used for dynamic monitoring. An essential parameter to measure the properties of a piezoelectric sensor is the piezoelectric coefficient. Materials used for piezoelectric sensors are usually divided into inorganic and organic classes, and the inorganic class includes barium titanate, zinc oxide, lead zirconate titanate, etc. They all have the disadvantage of poor flexibility while organic materials such as PVDF and PP are usually highly flexible but not conductive enough [111]. Therefore, additives are often used to enhance their piezoelectric properties. Moreover, the high electric voltage field during the electrospinning preparation is very favorable for the polarization of the piezoelectric material.

### 4.4. Triboelectric Effect

Triboelectric sensors can transform external mechanical signals into triboelectric signals through electrostatic and triboelectric effects. The basic working principle is that as two different non-electric substances come into contact with each other, the charge transfer will occur, generating a positive and negative electrostatic charge [112]. When the contact surfaces are separated, a voltage difference is created between the surfaces of these two materials, and the circuit formed by the wires between the two materials generates an electric current. Triboelectric sensors have the advantages of being self-powered, high instantaneous power, etc. However, the output performance is always affected by the amplitude and frequency of the mechanical stimulation. In order to relatively improve the output performance, researchers have now developed flexible triboelectric sensors with special structures and functions. As triboelectric sensors only generate an electrical signal when subjected to mechanical stimulation, they are only suitable for dynamic sensing, the same as piezoelectric sensors [113,114]. Moreover, signal interference generated by external parameters such as humidity and temperature variations is also an obvious problem to be solved in its practical application.

## 5. Application of the Electrospinning Nanofibers Based Artificial Skins

In recent years, various artificial skins have been reported successively and have played an important role in healthcare [115,116,117,118,119,120,121,122,123,124,125], HMI, and other fields [126,127,128,129,130,131,132,133]. Compared with other traditional electronic sensors, artificial electronic skin can meet the demand of human health monitoring and HMI when it is used in seamless and stable contact with human skin and obtains low impedance physiological signals [134,135,136,137,138]. Therefore, it has higher requirements on material permeability, tensile resistance, and biocompatibility [139]. Due to its high porosity, high toughness, and small mass, electrospun nanofiber-based bioinspired artificial skins with high flexibility and a three-dimensional porous mesh structure are often considered as the first choice [140,141,142,143,144,145,146,147,148,149,150].

### 5.1. Healthcare Monitoring

**Figure 7:**
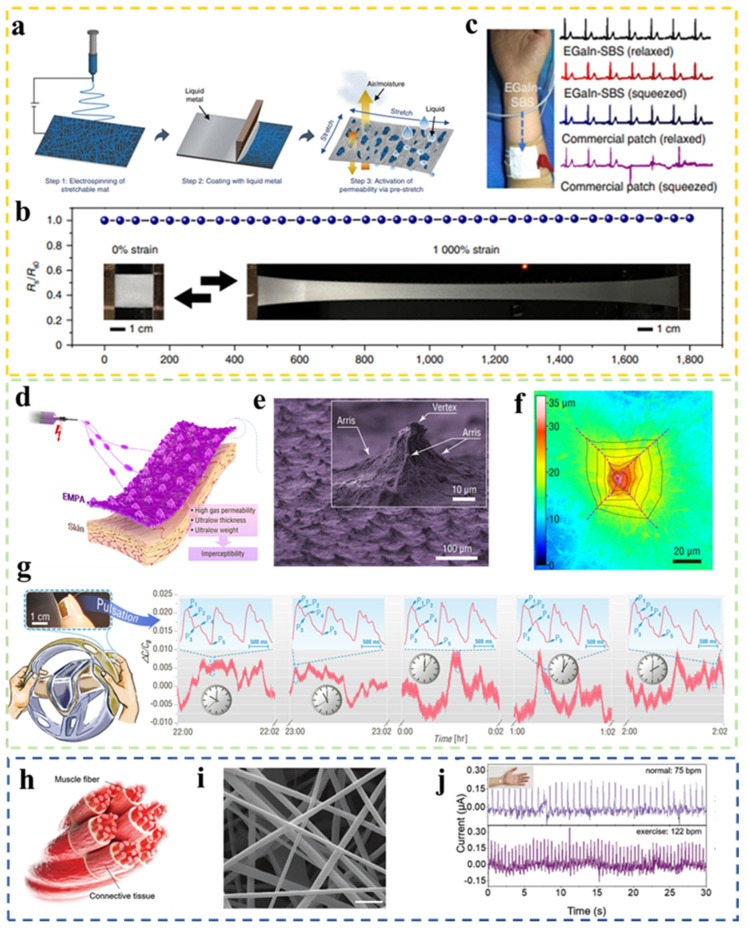
(**a**) The manufacturing procedure illustration of LMFM. (**b**) Impedance change of EGaln-SBS against tensile strain; the insets in b show EGaIn-SBS at 0% and 1000% strain, respectively. (**c**) ECG signal from ECG electrodes made by EGlan-SBS [171]. Copyright 2021 Springer Nature. (**d**) Schematic diagram of the manufacture and structure of EMPAs. (**e**) SEM image of EMPAs; the inset shows an enlarged SEM image of a single electrospun micropyramid. (**f**) LCM image of an electrospun micropyramid. (**g**) Sensors based on EMPAs manufactured to monitor a driver’s fingertip pulses for extended periods of time; the insets show magnified fingertip pulse waveforms. Copyright 2022 Springer Nature. (**h**) Schematic of muscle fibers in human leg [172]. (**i**) SEM image of electrospun fibers via dopamine coating. (**j**) Real-time pulse signals for static and after motion states [173]. Copyright 2021 Wiley-VCH.

With the advancement of flexible electronics, electrospun nanofiber-based artificial skin is increasingly being used for human health monitoring [151,152,153,154,155,156,157,158,159,160,161,162]. Comfortable, accurate, and real-time collection of physiological electrical signals is important for determining human health conditions. After prolonged conformal contact with human skin or joints, the ability to avoid elevated impedance caused by sweat and to withstand repeated mechanical deformation are crucial issues that need to be addressed [163,164,165,166,167,168,169,170]. Therefore, a flexible conductor with high permeability and stretchability (liquid-metal fiber mat, LMFM) was developed by Zheng et al. in 2021 [171]. The preparation is based on the coating of liquid metal (eutectic gallium-indium alloy, EGaln) on an electrospun fiber mat (styrene-butadiene-styrene, SBS). After the pre-stretching process, the liquid metal between the elastomeric SBS nanofibers formed a lattice-like structure and the LMFM maintained a high permeability to both gases and liquids (Figure 7a). In tensile tests, EGaIn-SBS can achieve over 1800% stretching while the conductor impedance remains at a low level without significant change during the process, showing ultra-high conductivity and electrical stability (Figure 7b). Therefore, an EGaIn-SBS containing three layers of printable gain electrodes was further developed that can be worn on the arm to acquire low-impedance ECG signals. As a result of its ultra-high elasticity, there is no significant change in signal waveform under stretch or compression compared to commercial ECG skin patches (Figure 7c). In addition, it can also realize the monitoring of sweat discharged from human skin, multimodal monitoring, and treatment of human skin by heating, and has a great application potential in medical monitoring.

In 2022, Pan et al. developed a self-assembly fabrication process for wearable devices based on wet heterostructure electrospinning technology [172]. Electrospinning micro-pyramidal arrays (EMPAs) with unique structures were constructed using a far-field electrospinning device with a charged grounded aluminum foil with bumps as the collector (Figure 7d). PVDF was used as the proof-of-concept material to fabricate the EMPAs-based films, and the SEM images showed a uniform planar distribution of the micro-pyramidal structure on the film, and typical features of the pyramidal structure were shown with the tilted three prongs intersecting at the apex (Figure 7e,f). Since the micro-pyramid structure microfibers constructed the permeable network, the film with EMPAs was ultra-thin, ultra-light, breathable, and suitable to be adopted as the artificial skin. Therefore, a piezoelectric capacitive sensor based on EMPAs was developed to collect pulse signals in real time for human health monitoring with high permeability and sensitivity. Figure 7g shows the clear obtained pulse peaks that reflect physiological indicators without any degradation of the signal waveform for four hours when a driver wore it for a long time without affecting normal work, which is of great importance for human health diagnosis. Similarly, inspired by human muscle fibers, Chen et al. proposed an electrospun fiber-based piezoelectric sensor which can capture human pulse signals for health monitoring (Figure 7h) [173]. Electrospun barium titanate/polyvinylidene fluoride (BTO/PVDF) nanofibers are modified mainly by using polydopamine (PDA). Groups of DA formed cross-links with the BTO nanoparticles due to van der Waals forces as well as attached to the PVDF polymer fibers, encasing the protruding BTO nanoparticles and making the fiber surface smooth. In addition, the piezoelectric performance had been greatly improved. Figure 7i shows the microscopic images of smooth nanofibers after 5 wt% PDA doping. After a simple encapsulation process, a piezoelectric fabric based on electrospun nanofibers was fabricated for monitoring pulse signals in real time. The ability of the device to monitor human health was verified by distinguishing weak changes in the pulse signals of the wearer in different states (Figure 7j).

**Figure 8:**
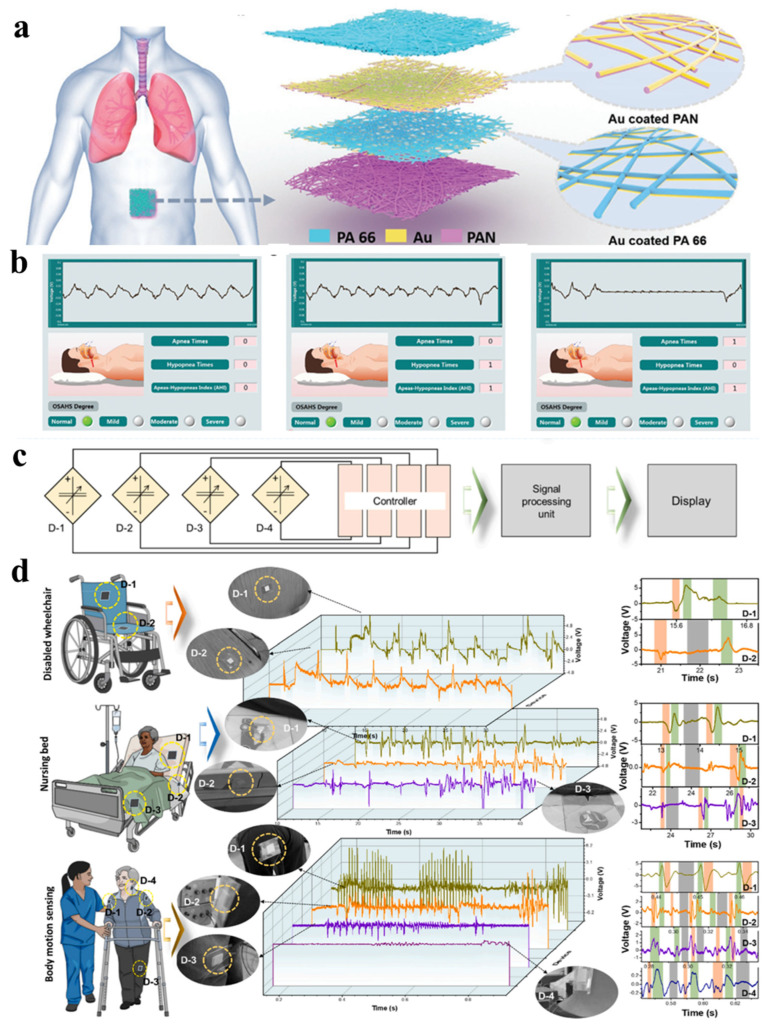
(**a**) An illustration of the structure of TENG-based SANES. (**b**) The sleep respiratory state judgment based on the OSAH diagnosis system [174]. Copyright 2021 Wiley-VCH. (**c**) Circuit diagram of health monitoring system connecting various TEHS devices to the monitor. (**d**) Integration of multiple TEHS in a wheelchair, nursing bed, and body for building health monitoring systems [175]. Copyright 2022 Elsevier.

In addition to myoelectric and pulse signals, electrospinning nanofiber-based artificial skin can also be used for several other health monitoring applications. For instance, Wang et al. developed a TENG-based nanofiber electronic skin (SANES) for respiratory monitoring and diagnosis during sleep (Figure 8a) [174], which was characterized by good permeability, high sensitivity, and was easy to wear. SANES is mainly assembled by the top encapsulation layer, middle functional layer, and bottom substrate layer, and all three nanofiber functional layers are prepared by electrospinning. The PA66 and PAN sandwiched in the middle were used as electrodes with a layer of Au of 100 nm thickness at the surface, respectively. The upper and lower parts are protected from electrode interference by PA66 and PAN as cover layers, respectively. The device is placed on the abdomen of the test subject, which monitors the occurrence of OSAHS during sleep based on the movement of the abdominal skin during breathing and records the number of apneas and hypoventilation states (Figure 8b). Classifying or alerting according to the severity has great application prospects in the area of personal sleep health monitoring. In addition to directly collecting physiological electrical signals for real-time monitoring of the human body, electrospun nanofiber-based devices can also monitor and provide early warnings of human health and safety by establishing medical monitoring systems. In 2022, Yu et al. prepared a triboelectric energy harvesting sensor (TEHS) using triboelectric fiber films made by electrospinning technology and built a medical monitoring system by multiple TEHS devices [175]. The system contains several sensors, a controller, a data processing unit, and a display unit (Figure 8c). Figure 8d shows the application of the system in a practical scenario, where the TEHS is mounted on a wheelchair, a nursing bed, and a human body to sense and monitor the human motion through the electrical response generated by the TEHS.

### 5.2. Intelligent HMI

**Figure 9:**
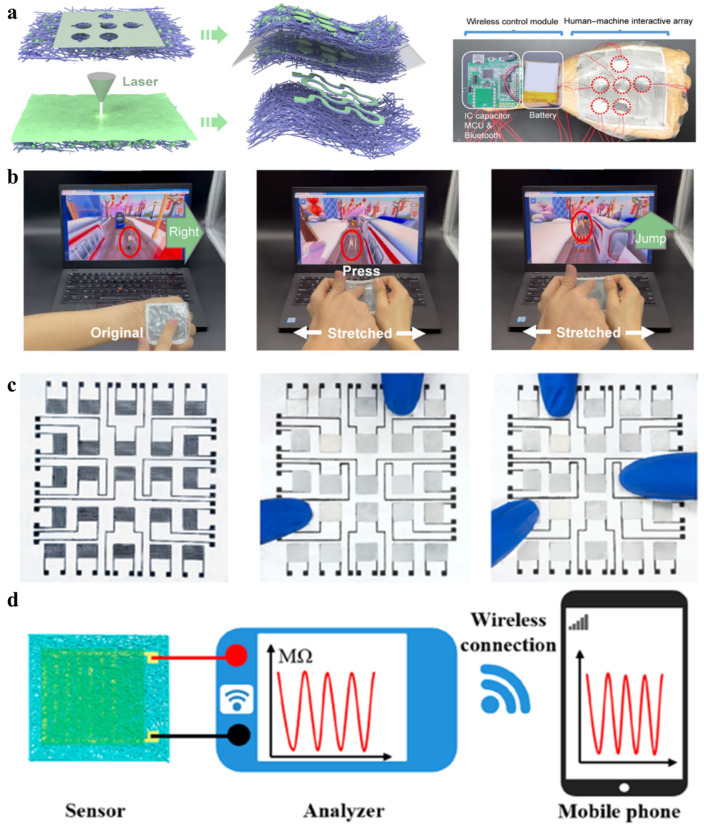
(**a**) Schematic illustration of NHSE-based electronic skin preparation and photos of the e-skin sensing array and wireless control module. (**b**) E-skin human-computer interaction system based on NHSE for playing computer games [208]. Copyright 2022 Wiley-VCH. (**c**) Images of pressure-sensing arrays based on MXene/protein composite nanofibers pressed by different numbers of fingers. (**d**) Concept illustration of wireless transmission [209]. Copyright 2021 American Chemical Society.

As artificial intelligence emerges and develops, artificial skin plays a crucial role not only for medical monitoring, but also for intelligent HMI [176,177,178,179,180,181,182,183,184,185,186,187,188,189,190,191]. Besides the monitoring of physiological parameters and the movement status of the human body, multifunctional artificial skin based on electrospun nanofibers can be used for mechanical control [192,193], on-demand therapy [194,195], and gesture recognition and intelligent control [196,197,198,199,200,201,202,203,204,205,206,207]. In 2022, a wearable flexible electrode (nano-liquid metal (LM)-based highly robust stretchable electrode, NHSE) that can be used for game control and thermal therapy was proposed by Li et al. [208]. Figure 9a illustrates the simple fabrication process, which crucially involves the manufacturing of mechanically robust and highly elastic nanofiber scaffolds by electrospinning polyurethane nanofibers, followed by electrospraying liquid metal (LM) nanoparticles into the TPU nanofiber scaffold to form a composite structure that simulates the interaction of water and web. LM provides electrical conductivity and the nanofiber scaffold provides the mechanical properties, achieving 500% tensile capacity without any additive binder. After 33,000 times of a 100% tensile cycle test, the impedance change is only 5%, showing high stability. Based on this, two NHSEs were partially activated and laser cut, then assembled to obtain a multifunctional artificial skin with a bilayer structure containing a capacitive sensor array and a wireless control unit. Figure 9b shows that the device was able to accurately recognize external signals and thus control the activities of the characters in the game under different conditions in tests, and it also enabled the input and recognition of numbers. It proves excellent potential in the area of HMI. Previously, Wan et al. have reported the development of MXene/protein nanocomposite fiber-based artificial skin for highly sensitive pressure sensing [209]. It can be used not only for disease diagnosis and motion detection, but also for human–machine interactive pressure detection. The individual pressure sensors are assembled by MXene impregnated silk protein nanofiber membranes and silk protein fiber membranes with MXene ink printed electrodes. When the external pressure increases in the operating range, the intensity of the generated current increases. Figure 9c shows an optical image of a 5 × 5 sensor array consisting of multiple sensors. Once the finger touches the sensor array, the magnitude and position of the applied pressure is quickly detected and sent to the user terminal via a wireless transmission module (Figure 9d).

**Figure 10:**
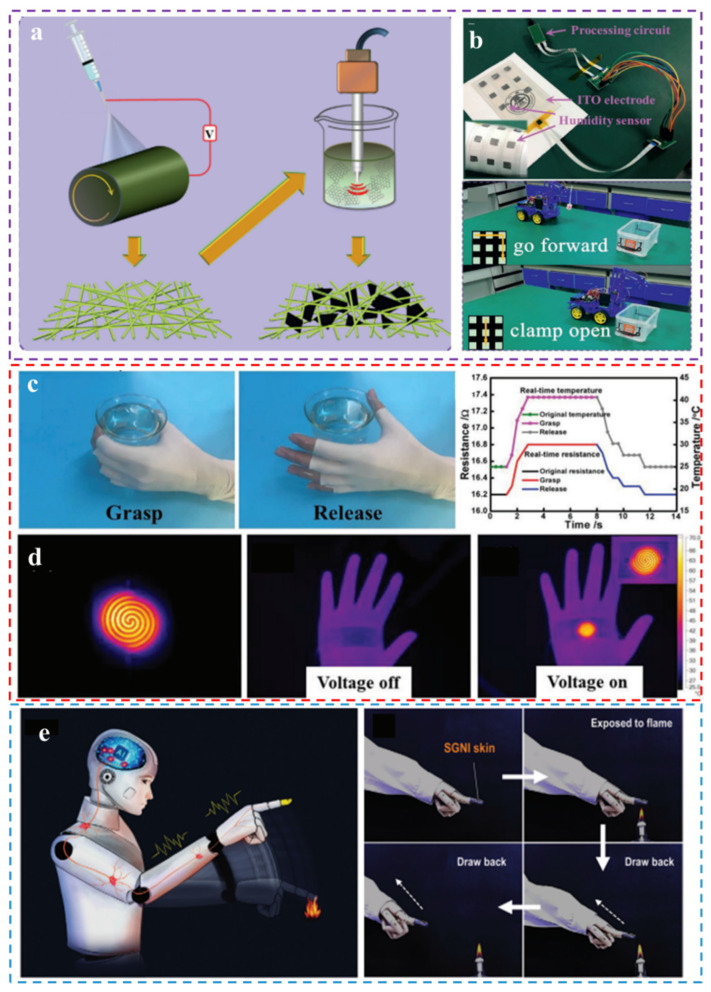
(**a**) Schematic diagram of manufacturing MPHS. (**b**) Photos of MPHS sensor array and smart cart driven by non-contact gestures [210]. Copyright 2021 Wiley-VCH. (**c**) Photographs of grasping and releasing a beaker, and the change in resistance and temperature signals obtained by the flexible heater when grasping and releasing a beaker containing hot water by hand. Copyright 2019 Wiley-VCH. (**d**) Images of the temperature distribution of the flexible heater when the applied voltage is 2.5 V and the temperature distribution attached to the human hand back before and after the DC voltage is applied [211]. (**e**) Schematic concept illustration for high temperature triggered protection system [212]. Copyright 2021 Wiley-VCH.

Different sensors have different working mechanisms; common flexible sensors used for HMI are not only pressure sensors, but also humidity sensors. During the COVID-19 epidemic, a flexible non-contact sensing array based on humidity sensing was reported by Yang et al. [210]. The single sensor (MG/PA66 humidity sensor, MPHS) is a composite material made of two-dimensional graphene flakes embedded in an electrospun PA66 nanofiber by ultrasonic treatment (Figure 10a). The characteristics of the electrospun nanofiber network give the composite a physical structure with a large ratio surface area, in addition to the PA66 chemical structure rich in water-absorbing functional groups, ensuring a high response to humidity. MPHS can be arranged to form a humidity sensing array for HMI in non-contact mode. As shown in Figure 10b, the sensing system for non-contact manipulation based on MPHS consisted of an MPHS, wireless transmission unit, analysis unit, and signal processing circuit. The motion control of the trolley can be achieved by non-contact sliding of the finger over the sensor array, indicating significant application prospects of this non-contact manipulation device in the public health field.

In addition to remote control, pressure detection, and game control, artificial skin made of electrospun nanofibers has extensive applications in areas such as healthcare and fire alarms. For example, Zhang et al. proposed a ventilatable artificial skin with real-time temperature monitoring and the ability to perform anti-infection heating therapy in 2019 [211]. The device consists of an electrospun moxifloxacin hydrochloride (MOX) nanofiber network with high toughness, gas permeability, and stability that can be used as a flexible heater when coated with a thermosensitive polymer film printed with a conductive pattern. Figure 10c shows real-time variations in resistance and temperature as the e-skin grasps and releases the beaker containing hot water, demonstrating the excellent thermal response of the e-skin. Figure 10d shows images of the temperature distribution of the e-skin when mounted on the human hand as a flexible heater, illustrating the ability of the electronic skin to be used as a flexible heater for human thermal therapy. Similar to this device, Ling et al. developed an electrospun flame retardant silk/graphene nanoionotronic e-skin (SGNI) with extremely high temperature sensitivity for integrating portable fire alarm systems [212]. When SGNI is exposed to a high temperature environment, the intelligent fire alarm system will sound an alarm and send an alarm message once the temperature reaches the alarm threshold. By using the ability of SGNI to respond to fire hazards, a concept was developed that could sense the position of the fire source and then control the robot to make evasive maneuvers in actual hazardous HMI situations (Figure 10e). Once the location of the robot installed on the SGNI skin is close to the fire source, the SGNI can sense the fire temperature and location within 6s and send signals back to the control unit to command the robot to actively avoid hazards and make a move away from the fire source, which provides highly promising applications in the area of secure and intelligent HMI.

## 6. Summary and Outlook

Due to the characteristics of high stretchability and superior porosity of electrospun nanofibers, they meet the demand of soft and air permeable artificial skin materials. As a result, electrospun nanofiber-based artificial skin has been rapidly developed and widely used. In this paper, we have reviewed the latest advances in electrospun nanofiber-based bioinspired artificial skin, mainly focusing on the electrospinning fabrication process and its influencing factors, materials selection and incorporation, sensing mechanisms of artificial skin, healthcare monitoring, and HMI application. A wide variety of abundant nanofibers have been designed and assembled by researchers through electrospinning by selecting suitable organic polymer solutions and process parameters. Therefore, flexible smart devices have made great progress in multifunctional sensing and bioapplicability, and many artificial skins with better performance have been developed based on them. In the future, with the enhancement of production technology, the application prospect of bioinspired artificial skin based on electrospun nanofibers will be more extensive. However, although significant advancements have been achieved in the preparation and design of various electrospun bionic skin devices, there are still many obvious issues remaining to be addressed and more work needs to be done in the following aspects.

### 6.1. Low Preparation Effectivity of Electrospun Artificial Skins

Bioinspired artificial skin based on electrospun nanofibers has low efficiency in the manufacturing process, including the fabrication of the spinning solution, electrospinning process, and nanofiber membrane for artificial skin assembly. For the current production technology, mass production on a large scale is difficult and costly to achieve. Such problems can only be solved with the development of production and manufacturing technologies.

### 6.2. Biosafety Issues of Bionic Artificial Skin

While the reported artificial skin tends to be fabricated by materials with good biocompatibility, there is still a long way to go before they can be truly used in the human body. As material science continues to advance, the issue of biosafety is a primary consideration for artificial skin.

### 6.3. Signal Interference Problem in Multi-Directional Detection

Although excellent progress has been obtained in unidirectional sensing of flexible bionic skin, the sensors produce similar signal changes when faced with external forces in different directions simultaneously. In practical applications, there are many situations where the force direction cannot be determined, so achieving effective identification and detection of multidirectional forces is still a challenge, and developing new materials or methods that can decouple multidirectional forces is a direction that future research needs to focus on.

### 6.4. The Processing of the Acquired Signals by Artificial Skin

In practical applications, the collected signals by the artificial skin on the human body will be interfered with by various factors, and the extraction of effective signals in a large number of signals is currently an important issue which should be tackled. The application of machine learning to assist in signal processing of artificial skin is an effective and promising solution for this field in the future.

### 6.5. System Integration of Artificial Skin Devices

As a crucial component in the field of flexible sensing for healthcare monitoring and HMI, how to integrate multiple modules (such as the data processing module, data transmission module, energy supply module, etc.) into the same platform is a big challenge for the current technology, which is a problem that must be solved for bioinspired artificial skin based intelligent systems to move toward more application scenarios.

Although there are still some urgent issues regarding the biosafety and application details of electrospun nanofiber-based bionic artificial skin, that does not take away from the fact that it is a promising manufacturing method for artificial skin. In the future, with the enhancement of production technology, the application prospect of bioinspired artificial skin based on electrospun nanofibers will be more extensive.

## Data Availability

Not applicable.
